# Orexin-2 Receptor Agonists in Narcolepsy: Restoring the Missing Signal

**DOI:** 10.3390/jcm15218642

**Published:** 2026-11-06

**Authors:** Yahya A. Alzahrani, Maan H. Harbi

**Affiliations:** 1 Department of Pharmacology, Faculty of Medicine, King Abdulaziz University, Rabigh 25732, Saudi Arabia; 2 Pharmacology and Toxicology Department, College of Pharmacy, Umm Al-Qura University, Makkah 21955, Saudi Arabia; mhiharbi@uqu.edu.sa

**Keywords:** narcolepsy, orexin, hypocretin, OX2R agonist, oveporexton, excessive daytime sleepiness, cataplexy, neuropharmacology

## Abstract

**Background/Objectives:** Narcolepsy type 1 (NT1) results from loss of hypothalamic orexin neurons. Selective orexin-2 receptor (OX2R) agonists now permit direct receptor-level restoration of deficient signaling, but recent broad reviews do not fully integrate the post-approval, trial-specific efficacy, prescribing, and safety evidence. This review asks what benefits and risks are supported by current data, which toxicities appear class-related versus molecule-specific, and how OX2R agonism should be positioned clinically. **Methods:** We performed a targeted narrative review of peer-reviewed human studies, major guidelines, trial registries, and regulatory documents updated through 16 October 2026. Peer-reviewed trials and regulatory sources were prioritized; conference- or sponsor-reported data were used only when peer-reviewed evidence was unavailable and were identified as provisional. **Results:** In the pivotal phase 3 trials, oveporexton produced placebo-adjusted improvements in MWT sleep latency of 13.8–20.1 min, ESS reductions of 8.0–9.8 points, and weekly cataplexy incidence-rate ratios of 0.25–0.38. Clinically important safety considerations include insomnia, urinary frequency/urgency, CPK elevations, CYP3A interactions, and measurable abuse potential. Alixorexton phase 2 data support OX2R agonism as a developing drug class, whereas firazorexton illustrates molecule-specific hepatotoxic risk. **Conclusions:** OX2R agonists are best described as mechanism-based pharmacological restoration of orexin signaling, not proven disease-modifying therapy. Their comparative clinical position remains uncertain because head-to-head trials with established therapies are lacking.

## 1. Introduction

Narcolepsy is a chronic disorder of sleep–wake regulation characterized by excessive daytime sleepiness (EDS), impaired maintenance of wakefulness, rapid eye movement (REM) sleep intrusions, disrupted nocturnal sleep, and, in NT1, cataplexy. Population estimates vary according to ascertainment and diagnostic method; a recent US general-population study estimated an overall narcolepsy prevalence of 37.7 per 100,000 persons, including 12.6 per 100,000 for NT1 [1]. The modern biological model of NT1 emerged from the discovery of the orexin (hypocretin) system in 1998 and subsequent genetic, animal, cerebrospinal fluid (CSF), and neuropathological evidence linking disruption of this pathway to the disorder [2,3,4,5,6,7,8].

Orexin-A and orexin-B are derived from prepro-orexin in a small population of lateral and perifornical hypothalamic neurons. Through OX1R and OX2R, these neurons project widely to histaminergic, noradrenergic, serotonergic, cholinergic, autonomic, and limbic networks and help stabilize wakefulness and REM-state boundaries [2,3,9,10,11]. The pathogenic relationship with NT1 is unusually direct: HCRTR2 mutations cause hereditary canine narcolepsy, orexin-deficient mice show sleep–wake instability and cataplexy-like arrests, and most patients with NT1 have profound orexin-neuron loss and very low CSF hypocretin-1 [4,5,6,7,8].

The burden of NT1 extends beyond sleep propensity to educational and occupational impairment, driving restrictions, psychiatric comorbidity, and reduced health-related quality of life [12,13]. Current guidelines support modafinil, pitolisant, sodium oxybate, solriamfetol, and selected stimulant or antidepressant strategies according to the dominant symptom and patient profile [14,15]. These therapies remain clinically important, but they act mainly through downstream catecholaminergic, histaminergic, or GABA-related pathways and may require combination treatment when multiple symptom domains persist.

Small-molecule OX2R agonists introduced a mechanistically different strategy by stimulating the receptor normally activated by endogenous orexin. This approach progressed from intravenous proof-of-mechanism studies to oral agents and, on 5 August 2026, to US Food and Drug Administration (FDA) approval of oveporexton (Orzeyful) for adults with NT1 [16]. Recent reviews, including the 2026 review by Kornum and colleagues, comprehensively summarize orexin biology and therapeutic opportunities [17]. The present review addresses a narrower post-approval gap by integrating peer-reviewed phase 3 evidence with label-level clinical pharmacology and safety and by quantitatively comparing the developing agonist class. Specifically, we ask (1) what magnitude of efficacy and risk is supported by trial-specific data; (2) which adverse effects appear class-related versus molecule-specific; and (3) how should direct OX2R agonism be positioned relative to established therapies and to true disease modification in the absence of head-to-head superiority data?

The biological origin of orexin deficiency also defines the limits of receptor agonism. NT1 has a strong HLA-DQB1*06:02 association and converging evidence of immune involvement, including orexin-reactive T-cell responses and environmental triggers, although the exact mechanism of neuronal loss remains debated [18,19,20,21]. A 2026 perspective emphasized that classical autoimmune destruction and alternative immune-associated models are not fully reconciled [19]. OX2R agonism can correct the downstream signaling deficit regardless of how neuronal loss arose, but it should not be assumed to halt the initiating process.

## 2. Methods: Evidence Identification and Appraisal

### 2.1. Search Strategy

We conducted targeted searches of PubMed/MEDLINE, primary journal websites, ClinicalTrials.gov, sponsor trial registries, and FDA regulatory documents from database inception through 16 October 2026. Search terms combined narcolepsy, orexin, hypocretin, orexin receptor 2, OX2R agonist, danavorexton/TAK-925, firazorexton/TAK-994, oveporexton/TAK-861, alixorexton/ALKS 2680, cleminorexton/ORX750, TAK-360, and ledasorexton/E2086. Reference lists of pivotal trials, guidelines, and recent reviews were also hand-searched to identify additional relevant publications.

### 2.2. Eligibility, Source Hierarchy, and Critical Appraisal

We included peer-reviewed human pharmacology and clinical trials of selective OX2R agonists; major narcolepsy treatment guidelines; pivotal randomized studies of established therapies used for clinical context; FDA or other regulatory documents required to verify approved dosing, pharmacokinetics, contraindications, adverse reactions, and regulatory status; and landmark mechanistic studies necessary to explain target biology. Duplicate reports were excluded. Animal-only studies were generally excluded unless they provided foundational target validation or directly informed receptor pharmacology. Sponsor press releases were not used when a peer-reviewed publication, regulatory document, or trial registry was available.

Evidence was weighted qualitatively by source hierarchy, study design, sample size, endpoint definition, and independence of the source. Peer-reviewed randomized trials and regulatory reviews were given greatest weight, whereas congress abstracts and company-reported results were treated as provisional and were not used as the sole basis for claims of comparative clinical superiority. For pivotal trials and directly relevant secondary analyses, we also examined endpoint completeness, early termination, the distinction between randomized and evaluable populations, missing-data handling, recognizable adverse effects that could threaten masking, selective or exploratory outcome reporting, and applicability to routine practice. These study-level limitations were incorporated into the narrative interpretation rather than treating randomization or peer review as sufficient evidence of reliability. Because this was a narrative rather than a systematic review, no formal risk-of-bias instrument or meta-analysis was applied. Recent broad reviews were used for context, whereas quantitative treatment claims were traced to primary or regulatory sources [17].

## 3. Biological and Therapeutic Context

### 3.1. Orexin Biology and the Rationale for Selective OX2R Agonism

Orexin-A and orexin-B derive from prepro-orexin and activate two G-protein-coupled receptors, OX1R and OX2R. Orexin-A has high affinity for both receptor subtypes, whereas orexin-B is relatively OX2R-selective [2,3,11]. Orexin neurons innervate major wake-promoting nuclei, including the tuberomammillary nucleus, locus coeruleus, dorsal raphe, and basal forebrain, and their distributed output contributes to sustained wakefulness and stabilization of transitions between wake, non-REM, and REM sleep [9,10,11,22].

OX2R is particularly important for wake-state stability. The canine HCRTR2 mutation, receptor-selective animal studies, and human pharmacological responses to danavorexton provide convergent support for OX2R as a therapeutically relevant node [4,23,24,25]. Structural work with TAK-925 has shown how small-molecule agonists engage and activate OX2R [24]. OX1R and OX2R have overlapping but non-identical functions; OX2R contributes strongly to sleep–wake continuity and histaminergic arousal, whereas OX1R has prominent roles in selected motivational, reward, and autonomic functions [9,10,11].

**Figure 1:**
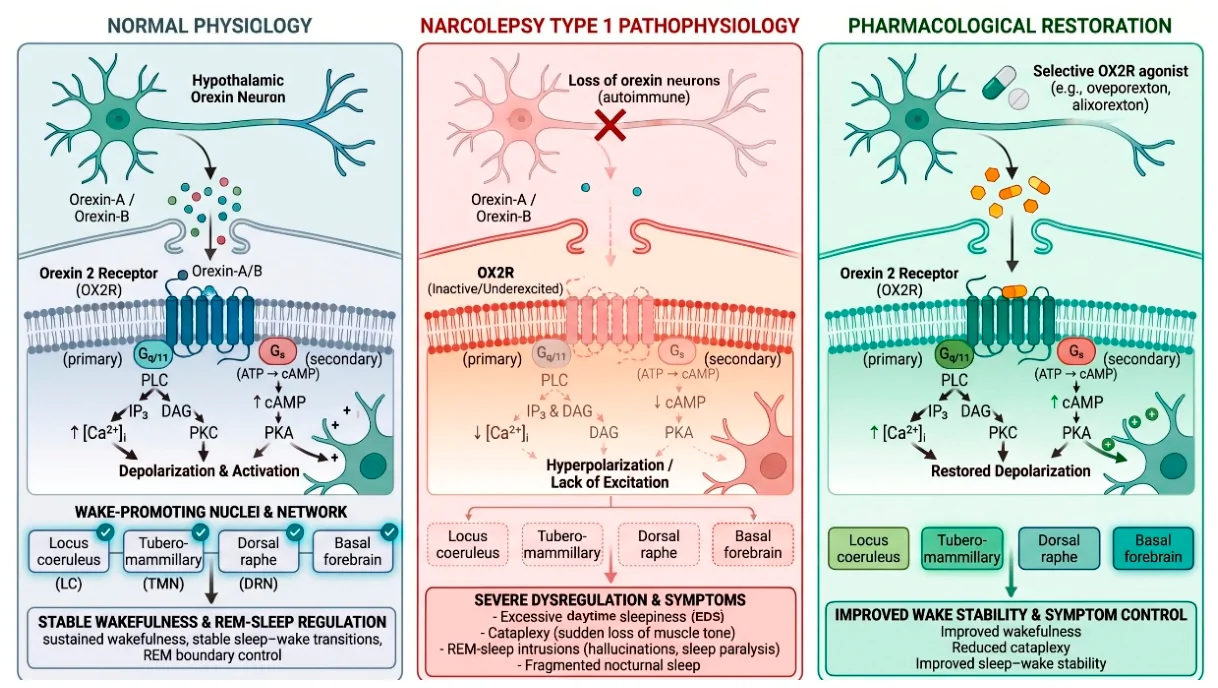
**Mechanistic rationale for OX2R agonism in narcolepsy type 1.** Under physiological conditions, hypothalamic orexin neurons release orexin-A and orexin-B, activating preserved OX2Rs within wake-promoting networks. OX2R activation engages intracellular signaling pathways, including Gq/11–phospholipase C (PLC)–inositol trisphosphate/diacylglycerol (IP_3_/DAG)–Ca^2+^/protein kinase C (PKC) signaling, with additional coupling to cAMP/protein kinase A (PKA), promoting neuronal excitation and stabilization of wake–REM boundaries. In NT1, loss of orexin neurons markedly reduces endogenous receptor stimulation and contributes to sleep–wake instability, excessive daytime sleepiness, cataplexy, REM-sleep intrusions, and fragmented nocturnal sleep. Selective OX2R agonists bypass the deficient endogenous ligand and directly activate preserved receptors, restoring downstream orexin-responsive signaling. This represents receptor-level functional restoration and should not be interpreted as neuronal regeneration or proven disease modification. **cAMP**, cyclic adenosine monophosphate; **DAG**, diacylglycerol; **DRN**, dorsal raphe nucleus; **EDS**, excessive daytime sleepiness; **IP_3_**, inositol trisphosphate; **LC**, locus coeruleus; **NT1**, narcolepsy type 1; **OX2R**, orexin-2 receptor; **PKA**, protein kinase A; **PKC**, protein kinase C; **PLC**, phospholipase C; **REM**, rapid eye movement; **TMN**, tuberomammillary nucleus.

The decisive pharmacological feature of NT1 is that the lesion is predominantly presynaptic: orexin-producing neurons are lost, while postsynaptic OX2Rs remain sufficiently responsive to exogenous stimulation (Figure 1). Human studies with danavorexton demonstrated marked wake-promoting effects in NT1 and NT2, directly supporting preserved receptor responsiveness [25]. OX2R signaling is not a simple on/off switch; receptor coupling can involve several G-protein families, calcium, phospholipase, kinase, and beta-arrestin pathways, creating opportunities for medicinal chemistry to alter potency, central exposure, duration, and potentially tolerability across compounds [11].

### 3.2. Established Pharmacotherapy: Practice-Oriented Context

Current treatment relies on pharmacologically distinct strategies. The American Academy of Sleep Medicine strongly recommends modafinil, pitolisant, sodium oxybate, and solriamfetol for adults with narcolepsy, with armodafinil, methylphenidate, and dextroamphetamine conditionally recommended; European guidance similarly emphasizes individualized selection according to symptom burden and patient characteristics [14,15]. Modafinil/armodafinil primarily influence dopaminergic wake pathways; solriamfetol inhibits dopamine and norepinephrine transporters; traditional stimulants amplify catecholaminergic signaling; pitolisant increases histaminergic transmission through H3-receptor inverse agonism; and oxybates improve EDS, cataplexy, and nocturnal sleep through broader GABA-related mechanisms.

Established randomized evidence supports meaningful symptomatic efficacy for these therapies: modafinil improves objective and subjective sleepiness, pitolisant improves EDS and reduces cataplexy, solriamfetol produces dose-related improvements in MWT and ESS, and oxybates improve multiple symptom domains [26,27,28,29,30,31]. OX2R agonism is mechanistically more proximal to the orexin deficit in NT1, but mechanistic proximity should not be interpreted as evidence of superior clinical effectiveness. No head-to-head randomized trial has established the superiority of an OX2R agonist over modafinil, solriamfetol, pitolisant, or an oxybate. The practical question is therefore whether direct OX2R agonism can deliver broad, durable symptom control with an acceptable treatment burden and safety profile (Table 1).

## 4. Clinical Development and Clinical Pharmacology of OX2R Agonists

### 4.1. Danavorexton: Human Proof of Mechanism

Danavorexton (TAK-925), a highly selective OX2R agonist, produced receptor-dependent wake promotion in preclinical models and helped define the structural basis of OX2R activation [23,24]. In human studies, intravenous danavorexton markedly increased MWT sleep latency in NT1 and NT2, confirming that OX2R remains pharmacologically responsive despite chronic orexin deficiency [25]. A randomized crossover study in idiopathic hypersomnia (IH), where CSF orexin is generally preserved, also showed a large increase in objective wakefulness after a single infusion [38]. These short intravenous exposures established proof of mechanism but cannot exclude delayed or idiosyncratic hepatotoxicity that might emerge only with longer oral exposure; they therefore should not be used to infer hepatic safety for chronic treatment.

### 4.2. Firazorexton (TAK-994): Target Validation, Molecular Failure

The orally active OX2R agonist firazorexton (TAK-994) showed pronounced efficacy signals in a randomized phase 2 NT1 trial (30, 90, or 180 mg twice daily for 8 weeks). Placebo-adjusted least-squares mean improvements in MWT sleep latency were 26.4, 29.9, and 35.0 min across the three doses; ESS differences versus placebo were −10.1, −11.4, and −13.0 points; and weekly cataplexy rate ratios were 0.05, 0.20, and 0.15, respectively [39]. However, the trial was terminated early because of hepatic adverse events, and primary-endpoint data were available for only 41 of 73 randomized participants (56%), with early termination accounting for most missing data. ESS and weekly cataplexy analyses were likewise incomplete (available for 58% and 53% of randomized participants, respectively) [39]. The magnitude of the observed effects therefore supports pharmacological target validation but should not be interpreted as a stable comparative efficacy estimate because substantial post-randomization missingness and premature termination reduce precision and may limit generalizability. Clinically important liver-enzyme elevations occurred in five participants, and three met Hy’s law criteria for drug-induced liver injury (DILI) [39]. Mechanistic toxicology subsequently found wide margins for direct cytotoxicity, mitochondrial toxicity, and bile-salt transport inhibition but identified metabolism-dependent covalent binding consistent with an idiosyncratic, molecule-specific liability [40]. Thus, TAK-994 provided important target-validation evidence while failing as a drug molecule, with both efficacy and safety findings interpreted in the context of the incomplete dataset.

### 4.3. Oveporexton: Pivotal Clinical Evidence

Oveporexton (TAK-861) is an orally active, potent, selective OX2R agonist with approximately 3000-fold in vitro selectivity for OX2R over OX1R in the FDA review [33]. Preclinical studies demonstrated OX2R-dependent wake promotion and improvement of narcolepsy-like phenotypes [41]. In the randomized phase 2b trial (TAK-861-2001; 112 adults with NT1; 8 weeks), oveporexton produced dose-related improvements in MWT sleep latency and ESS and reduced weekly cataplexy; insomnia and urinary symptoms were common, while no TAK-994-like hepatotoxicity signal was detected during the trial [34].

#### Phase 3 Evidence and 2026 Approval

Two pivotal 12-week, randomized, double-blind, placebo-controlled trials followed. First Light (NCT06470828) randomized 168 participants to placebo, oveporexton 1 mg twice daily, or 2 mg twice daily; Radiant Light (NCT06505031) randomized 105 participants to placebo or 2 mg twice daily [32,33]. The primary endpoint was change in mean MWT sleep latency at week 12, with ESS, weekly cataplexy rate (WCR), Narcolepsy Severity Scale for Clinical Trials, and patient global measures among prespecified secondary endpoints. Both trials met their primary and multiplicity-controlled secondary efficacy endpoints [32].

Trial-specific estimates are more informative than mixing pooled and individual-trial analyses. In First Light, placebo-adjusted MWT improvements were 13.8 min (95% CI 10.2–17.4) with 1 mg twice daily and 17.2 min (13.7–20.7) with 2 mg twice daily; in Radiant Light, the corresponding difference with 2 mg twice daily was 20.1 min (16.6–23.6) [32,33]. Placebo-adjusted ESS differences were −8.0, −9.8, and −9.5 points, respectively. WCR incidence-rate ratios were 0.34 (0.20–0.57), 0.38 (0.23–0.61), and 0.25 (0.15–0.42), corresponding to 66%, 62%, and 75% relative rate reductions versus placebo, not the within-treatment percentage reductions from baseline [33]. At week 12, MWT sleep latency ≥ 20 min was achieved by 48%, 56%, and 69% of the respective active groups (versus 6% and 0% in the corresponding placebo groups), while ESS ≤ 10 was achieved by 67%, 83%, and 84% (versus 17% and 12%) [32]. These thresholds describe patient-level benchmark attainment and should not be equated with restoration of normal sleep–wake physiology (Table 2). A separate pooled congress analysis reported WCR incidence-rate ratios of 0.28 and 0.32, equivalent to 72% and 68% relative rate reductions versus placebo; these estimates are analytically distinct from the 79.0–88.8% median within-treatment reductions from baseline reported elsewhere [42]. The pivotal trials provide strong short-term internal evidence, but their 12-week controlled duration, protocol-driven medication washout, selected eligibility criteria, and limited characterization of older adults and patients with severe organ impairment constrain direct extrapolation to long-term routine practice [32,33].

Phase 3 congress abstracts report improvements in cognitive measures, narcolepsy-specific functioning, REM-sleep architecture, sleep paralysis, hallucinations, and disturbed nighttime sleep [35,36,37]. These reports remain abstract-level secondary or exploratory evidence. Importantly, cognition has also been examined in a separate peer-reviewed secondary analysis of the phase 2 TAK-861-2001 trial. In that 8-week dataset, oveporexton improved attention (Psychomotor Vigilance Task), visual associative memory, and executive-function measures versus placebo [43]. Because these cognitive tests were exploratory outcomes and the trial was not powered specifically for cognition, they strengthen but do not establish an independent cognitive treatment effect. The investigators explicitly noted that larger studies are required to determine whether cognitive improvement reflects direct OX2R-mediated effects or is partly secondary to reduced sleepiness [43]. The phase 2 peer-reviewed cognitive analysis and the later phase 3 congress abstracts therefore represent different datasets and are not pooled or treated as interchangeable evidence; the former has higher publication maturity, whereas the latter provides supportive replication that remains provisional.

On 5 August 2026, the FDA approved oveporexton (Orzeyful) for adults with NT1, making it the first approved OX2R agonist [16]. China approved oveporexton for patients aged 16 years and older on 22 July 2026, and Japan approved it for adults on 24 August 2026; these regulatory milestones are cited from official manufacturer announcements because English-language regulatory documents were not readily accessible [44,45]. The significance of approval is therefore pharmacological and regulatory: direct OX2R agonism is now clinically viable, but this does not prove that the class is clinically superior to existing narcolepsy therapies.

### 4.4. Clinical Pharmacology and Drug Interactions

The FDA-approved regimen is either 1 mg after awakening followed by 1 mg 3–5 h later or 2 mg after awakening followed by 2 mg 3–5 h later, with a maximum of 4 mg/day [33]. The pivotal trials used two morning doses at least 3 h apart, with the second dose taken no later than 1 PM [33]. This split schedule reproduces the studied exposure pattern and daytime pharmacodynamic coverage; the available evidence does not establish that a split dose is mechanistically required or that a single morning dose would provide equivalent efficacy. Oveporexton reaches a median Tmax of approximately 1.5 h (range 1–4), has a mean terminal half-life of 23.2 h, reaches steady state within 5 days, and is >96% protein-bound [33]. Thus, early-day dosing reduces late-day peak exposure but does not eliminate evening drug exposure or guarantee freedom from insomnia—an important qualification given the high early incidence of insomnia in the pivotal trials. Metabolism is predominantly via CYP3A4. Strong CYP3A inhibitors are contraindicated; moderate inhibitors require reduction to 0.5 mg twice daily; and strong or moderate CYP3A inducers should be avoided. Itraconazole increased AUC 7.4-fold, fluconazole increased AUC 2.9-fold, and phenytoin decreased AUC by 81% [33]. Severe hepatic impairment (Child–Pugh C) and severe renal impairment requiring dialysis were not studied, and use is avoided in these populations [33].

### 4.5. Safety: Class Effects Versus Molecule-Specific Toxicity

The development history of the class permits a useful, but still provisional, distinction between molecule-specific and potentially mechanism-related adverse effects. Firazorexton-associated DILI was clinically serious, but transcriptomic/proteomic database analyses cited in the mechanistic toxicology study did not detect OX1R or OX2R in human hepatic or immune tissues, and experimental work found wide safety margins for several intrinsic hepatotoxic mechanisms [40]. Metabolism-dependent covalent binding provided a more plausible explanation for idiosyncratic TAK-994 injury [40]. The absence of a comparable signal in the 12-week oveporexton phase 3 program supports—without proving—the interpretation that TAK-994 hepatotoxicity was molecule-specific. Conversely, the short single- or limited-dose intravenous danavorexton studies cannot exclude a delayed idiosyncratic hepatic liability [25,38].

Urinary and sleep-related effects recur across chemically distinct OX2R agonists and are more consistent with on-target pharmacology. According to pooled pivotal oveporexton safety data, insomnia occurred in 55% of participants receiving 1 mg twice daily and 60% receiving 2 mg twice daily versus 1% with placebo; urinary frequency occurred in 53%, 58%, and 5%, respectively, and urinary urgency in 15%, 16%, and 1% [33]. Approximately 90% of insomnia events began within the first 2 days, 63% resolved within 1 week, but about 17% remained ongoing at study completion [33]. CPK elevations > 5× the upper limit of normal occurred in 11% of oveporexton-treated participants versus 5% with placebo; two participants with marked CPK and transaminase elevations discontinued treatment, although myoglobinuria and renal impairment were not observed [33]. Day-1 increases in systolic or diastolic blood pressure > 20 mmHg occurred in 22% and 9% of treated participants versus 13% and 7% with placebo, and heart-rate increases >15 beats/min occurred in 30% versus 22%; by week 12, placebo-adjusted mean changes were <5 mmHg for blood pressure and <2 beats/min for heart rate, with no significant week-10 ambulatory differences [33]. The label also reports LDL cholesterol > 160 mg/dL in 32% of treated participants versus 20% with placebo, supporting continued metabolic as well as cardiovascular surveillance [33]. These frequencies have direct prescribing implications. Patients should be counselled about insomnia, with dose reduction or discontinuation considered when persistent insomnia materially impairs daytime functioning or quality of life; lower urinary tract symptoms should be assessed before treatment and patients with overactive bladder monitored for worsening symptoms; and patients should be advised to report unexplained muscle pain, weakness, or dark urine, particularly during vigorous exercise or when using concomitant drugs associated with myotoxicity [33]. The marked imbalance in recognizable insomnia and urinary effects also creates a plausible risk of functional unblinding. No formal assessment of treatment-guess accuracy was reported, so subjective outcomes such as ESS and patient-reported global or functional measures should be interpreted with this potential bias in mind. This concern is less likely to account for the substantial objective MWT effects, which provide an important counterweight to possible expectancy effects [32,33].

Abuse liability should be interpreted from clinical data rather than regulatory scheduling alone. In a human abuse-potential study in recreational CNS-stimulant users, supratherapeutic oveporexton doses produced Drug-Liking scores greater than placebo and similar to or lower than phentermine 75 mg; euphoria was reported in 0–4.2% of oveporexton exposures versus 4.1% with phentermine and none with placebo [33]. Among 277 participants monitored after repeat-dose studies, the label reports limited evidence of withdrawal-related adverse events suggesting physical dependence [33]. These observations support risk assessment and monitoring but do not justify either dismissing or overstating misuse potential.

### 4.6. The Emerging OX2R Agonist Pipeline

**Figure 2:**
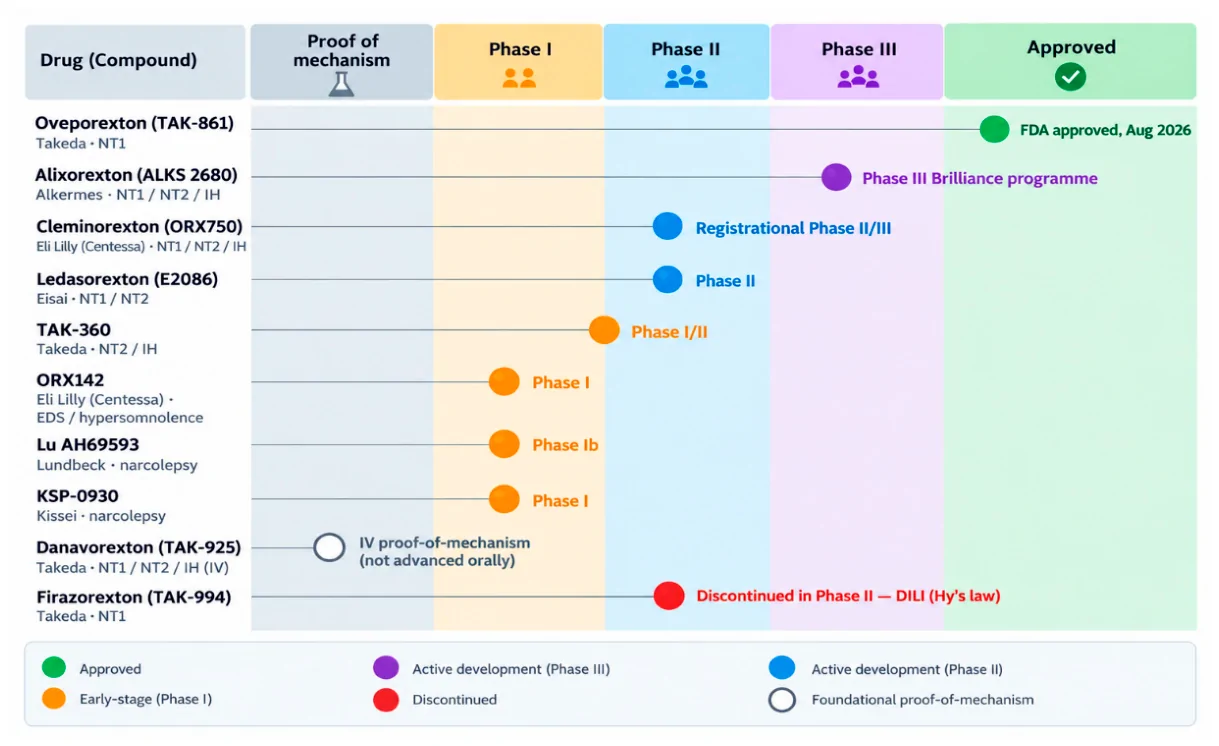
Clinical development of selected OX2R agonists as of October 2026. The figure shows the highest publicly verifiable development stage of selected OX2R agonists, including approved therapy, active phase 3, phase 2/3, phase 2, and phase 1 programs, foundational intravenous proof-of-mechanism work, and discontinued development. Sources for the development stage: oveporexton [16,32,33]; alixorexton [47]; cleminorexton [49]; ledasorexton [50]; TAK-360 [51,52]; ORX142 [53]; Lu AH69593 [54]; KSP-0930 [55]; danavorexton [25,38]; and firazorexton [39,40]. Development-stage position is descriptive and should not be interpreted as evidence of comparative efficacy or safety. DILI, drug-induced liver injury; IH, idiopathic hypersomnia; IV, intravenous; NT1/NT2, narcolepsy type 1/type 2; OX2R, orexin-2 receptor.

Approval of oveporexton established regulatory viability, but class differentiation will depend on dosing convenience, breadth of indication, efficacy, and safety (Figure 2; Table 3). Alixorexton (ALKS 2680; Alkermes, Dublin, Ireland) is an oral OX2R agonist evaluated once daily in the peer-reviewed phase 2 Vibrance-1 trial. Among 92 adults with NT1, placebo-corrected MWT changes at week 6 were 22.2 min (95% CI 17.2–27.2), 24.1 min (19.0–29.1), and 26.0 min (21.0–31.0) with 4, 6, and 8 mg, respectively [46]. Common treatment-emergent adverse events included pollakiuria (55%), insomnia (28%), salivary hypersecretion (25%), micturition urgency (14%), and blurred vision (14%) [46]. Vibrance-2 extended evaluation to NT2 and was reported as a congress abstract, while the phase 3 Brilliance program is ongoing [47,48]. These non-NT1 data are important mechanistically but remain less mature than the peer-reviewed NT1 evidence.

Cleminorexton (ORX750), ledasorexton (E2086), and TAK-360 remain investigational. Cleminorexton is being evaluated in the registrational SAPPHIRE phase 2/3 trial in NT1 and NT2 [49], while ledasorexton remains in phase 2 development [50]. TAK-360 is in phase 2 development, including registered trials in NT2 and idiopathic hypersomnia [51,52]. Earlier-stage OX2R programs include ORX142 (Centessa), which is being evaluated in a phase 1 first-in-human study in healthy adults that includes a sleep-deprivation proof-of-concept component [53]; Lu AH69593 (Lundbeck), which is being evaluated in a phase 1 study including participants with narcolepsy and is described by the sponsor as being in phase 1b development [54]; and KSP-0930 (Kissei), which entered a randomized phase 1 study in healthy volunteers in 2026 [55]. No mature patient-level efficacy data are currently available for these early-stage agents. Accordingly, Table 3 distinguishes peer-reviewed human efficacy evidence from registry or sponsor-reported development-stage information, and Figure 2 reports the highest publicly verifiable stage as of October 2026.

## 5. Clinical Interpretation and Positioning

### 5.1. Functional Replacement, Mechanism-Based Therapy, or Disease Modification?

“Orexin replacement therapy” is intuitive but pharmacologically imprecise. OX2R agonists do not replace lost neurons, supply native orexin-A or orexin-B, reverse immune-associated neuronal loss, or regenerate the orexin system. They provide a synthetic signal at a receptor normally engaged by endogenous orexin. “Pharmacological restoration of orexin signaling” or “functional receptor-level restoration” therefore better describes the intervention: the missing physiological input is functionally substituted, but the molecule is a receptor agonist rather than a peptide or cellular replacement.

**Figure 3:**
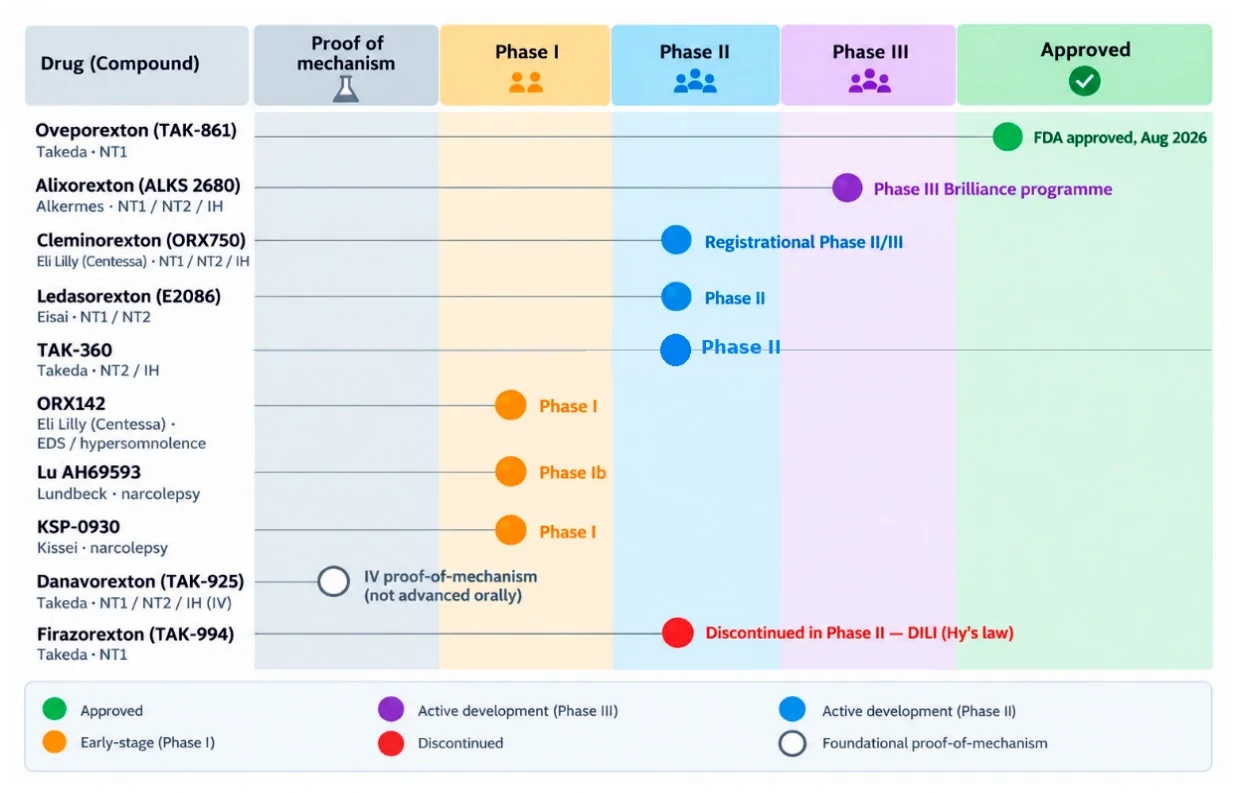
Therapeutic positioning of OX2R agonists in narcolepsy. Four conceptual tiers—symptom-directed therapy, network-level modulation, mechanism-based restoration, and (future) disease modification—are arranged by biological proximity to the orexin-signaling defect. Selective OX2R agonists provide mechanism-based therapy with functional pathway restoration; this is not proven disease modification, and greater mechanistic proximity does not establish superior clinical effectiveness. OX2R, orexin-2 receptor.

The same distinction determines whether the class should be called disease-modifying. Current evidence does not show preservation of surviving orexin neurons, prevention of the initiating process, neuronal regeneration, or persistence of benefit after treatment withdrawal. OX2R agonists are therefore mechanism-based symptomatic therapies with functional pathway restoration, not proven disease-modifying therapies. Furthermore, greater mechanistic proximity to the orexin deficit does not establish superior clinical effectiveness over established drugs; such a claim requires direct comparative trials (Figure 3).

### 5.2. NT2 and Idiopathic Hypersomnia: Replacement or Amplification?

Evidence outside NT1 challenges a purely replacement-based interpretation. In NT1, profound orexin deficiency provides a direct rationale for receptor agonism; in NT2 and IH, CSF orexin concentrations are generally normal or intermediate, although both disorders are heterogeneous [56,57]. Danavorexton improved objective wakefulness in IH [38], and alixorexton has shown wake-promoting effects in NT2 in early clinical development, including congress-reported phase 2 data [48]. These observations test whether OX2R agonism requires ligand deficiency or can enhance wake stability despite preserved bulk orexin.

Several mechanisms could explain benefit without overt orexin deficiency. A normal bulk CSF concentration does not guarantee normal temporal release, receptor engagement, or downstream signaling during high wake demand; alternatively, selective OX2R agonism may provide pharmacological amplification of an otherwise intact arousal network. The class may therefore act as functional receptor-level replacement in orexin-deficient NT1 and as wake-circuit amplification in NT2 or IH. Future studies should test whether exposure–response relationships, dose timing, receptor occupancy, and adverse effects differ according to phenotype and endogenous orexin status rather than assuming that the optimal NT1 regimen transfers unchanged to other hypersomnolence disorders.

### 5.3. Clinical Positioning and Research Priorities

The ultimate position of OX2R agonists in treatment algorithms remains uncertain. Placebo-controlled efficacy is substantial, but direct comparison with modafinil, solriamfetol, pitolisant, and oxybates is needed before claims of superiority, treatment replacement, or routine first-line preference can be made. Comparative-effectiveness studies should evaluate patient-relevant outcomes, including driving-related performance, cognitive endurance, work or academic functioning, nighttime sleep, quality of life, treatment burden, preference, and the ability to simplify multidrug regimens, not only MWT, ESS, and cataplexy frequency [12,13,37].

Three research priorities follow from the current evidence. First, long-term extension and real-world studies must establish durability over years and quantify persistent insomnia, urinary effects, CPK elevations, cardiovascular/metabolic signals, uncommon hepatic events, and outcomes in older adults, pregnancy, hepatic or renal disease, and pediatric narcolepsy. Second, pragmatic head-to-head and sequencing studies should determine whether OX2R agonists replace, reduce, or add to current polypharmacy and should incorporate functional and safety outcomes important to patients. Third, biomarker- and phenotype-guided studies, including CSF hypocretin, digital sleep–wake measures, electrophysiological markers, or receptor-occupancy approaches, should define who benefits across the NT1–NT2–IH spectrum and whether mechanism-based symptom restoration can eventually be combined with immune, neuroprotective, or regenerative strategies that truly alter disease biology.

## 6. Conclusions and Future Perspectives

Selective OX2R agonists represent a major translation of orexin biology into mechanism-based clinical pharmacology. Danavorexton demonstrated that preserved OX2R can be pharmacologically activated in humans; firazorexton validated oral efficacy while revealing a serious molecule-specific hepatic liability; and oveporexton established phase 3 efficacy and regulatory viability. The most defensible interpretation is pharmacological restoration of orexin signaling at the receptor level—not neuronal replacement, proven disease modification, or demonstrated superiority over established narcolepsy therapies. Post-approval practice should therefore balance the magnitude of trial-specific efficacy against frequent insomnia and urinary effects, CPK and metabolic monitoring signals, CYP3A interactions, and measurable abuse potential.

Future progress should focus on three priorities: long-term safety and durability in routine care; head-to-head comparative-effectiveness studies that include functional outcomes and treatment burden; and phenotype- or biomarker-guided expansion beyond NT1 while pursuing true disease-modifying approaches in parallel. These questions will determine whether OX2R agonism becomes a replacement for current multidrug strategies, an adjunct to them, or one component of a broader precision-treatment framework for central hypersomnolence.

## Figures and Tables

**Table 1:** Conventional narcolepsy pharmacotherapy compared with direct OX2R agonism.

Treatment/Class	Principal Action	EDS	Cataplexy	Nocturnal Symptoms	Relationship to Orexin Deficit	Main Practice Limitation	Relevant References
Modafinil/armodafinil	DAT-related wake-network modulation	Yes	Limited	Limited	Downstream; does not directly stimulate OX2R	Residual symptoms; interactions	[14,15,26]
Solriamfetol	DAT/NET inhibition	Yes	No established direct effect	Limited	Downstream; non-orexin target	Blood-pressure/heart-rate effects	[14,15,29]
Stimulants	Catecholaminergic potentiation	Yes	Limited	No	Downstream; non-orexin target	Cardiovascular and misuse risk	[14,15]
Pitolisant	H3 inverse agonism	Yes	Yes	Variable	Downstream; non-orexin target	Interactions; variable response	[14,15,27,28,31]
Oxybates	GABA-related modulation; sleep consolidation	Yes	Yes	Yes	Downstream; non-orexin target	Dosing burden; CNS-depressant precautions	[14,15,30]
Oveporexton (OX2R agonist)	Direct selective OX2R agonism	Yes	Yes	Provisional improvement (congress abstracts)	Direct receptor agonism; functional signalling restoration without neuronal replacement	Insomnia; urinary effects; CPK elevation; CYP3A interactions	[32,33,34,35,36,37]

Abbreviations: CNS, central nervous system; CPK, creatine phosphokinase; DAT, dopamine transporter; EDS, excessive daytime sleepiness; GABA, gamma-aminobutyric acid; NET, norepinephrine transporter; OX2R, orexin-2 receptor.

**Table 2:** Trial-specific phase 3 efficacy estimates for oveporexton in narcolepsy type 1.

Study/Active Dose	*N*/Duration	MWT: LS Difference vs. Placebo (95% CI)	ESS: LS Difference vs. Placebo (95% CI)	WCR at Week 12; IRR vs. Placebo (95% CI)	Patient-Level Week-12 Benchmark	Relevant References
First Light—1 mg BID	61 randomized; 12 wk	+13.8 min (10.2–17.4)	−8.0 (−9.9 to −6.1)	9.0/week; IRR 0.34 (0.20–0.57)	MWT ≥ 20 min: 27/56 (48%) vs. 2/36 (6%) placebo; ESS ≤ 10: 38/57 (67%) vs. 6/36 (17%)	[32,33]
First Light—2 mg BID	66 randomized; 12 wk	+17.2 min (13.7–20.7)	−9.8 (−11.6 to −7.9)	12.6/week; IRR 0.38 (0.23–0.61)	MWT ≥ 20 min: 35/62 (56%) vs. 2/36 (6%) placebo; ESS ≤ 10: 53/64 (83%) vs. 6/36 (17%)	[32,33]
Radiant Light—2 mg BID	70 randomized; 12 wk	+20.1 min (16.6–23.6)	−9.5 (−11.1 to −8.0)	7.1/week; IRR 0.25 (0.15–0.42)	MWT ≥ 20 min: 46/67 (69%) vs. 0/34 (0%) placebo; ESS ≤ 10: 56/67 (84%) vs. 4/34 (12%)	[32,33]

Values are trial-specific estimates from the peer-reviewed phase 3 publication and US prescribing information; pooled conference estimates are intentionally not mixed with these rows. Randomized group sizes are shown separately from the evaluable denominators used for the week-12 benchmark proportions. The benchmark *n*/*N* values are observed in evaluable participants and therefore should not be interpreted using the randomized denominator. MWT and ESS least-squares mean comparisons used the prespecified longitudinal models with placebo-based multiple imputation for missing data; WCR values show the absolute week-12 mean rate and incidence-rate ratio versus placebo. Patient-level benchmark percentages indicate attainment of MWT ≥ 20 min and ESS ≤ 10 and do not imply restoration of normal sleep physiology [32,33]. Abbreviations: BID, twice daily; CI, confidence interval; ESS, Epworth Sleepiness Scale; MWT, Maintenance of Wakefulness Test; WCR, weekly cataplexy rate.

**Table 3:** Standardized cross-agent evidence summary for selected OX2R agonists.

Agent (Code)	Study/Population/Exposure	Efficacy Versus Placebo (Standardized Domains)	Safety Context	Evidence Limitations/Status
Danavorexton (TAK-925)	Human IV proof-of-mechanism studies; NT1/NT2/IH; acute or single-infusion exposure	MWT: marked acute wake-promoting effect; protocols/exposure differ across studies. ESS: NR. Cataplexy: NR.	Limited-dose IV exposure; urinary effects reported	Foundational peer-reviewed proof of mechanism; exposure too short to establish chronic hepatic safety [25,38]
Firazorexton (TAK-994)	Randomized phase 2 NT1; *n* = 73; 30/90/180 mg BID; planned 8 wk, terminated early	MWT: +26.4/+29.9/+35.0 min; primary endpoint available in 41/73 (56%). ESS: −10.1/−11.4/−13.0 points; available in 58%. Cataplexy: rate ratios 0.05/0.20/0.15; available in 53%.	Any AE: 44/56 (79%) active vs. 4/17 (24%) placebo; 5 liver-enzyme cases; 3 Hy’s law cases	Large effects but substantial missingness from early termination; discontinued for DILI [39,40]
Oveporexton (TAK-861)	Two randomized phase 3 NT1 trials; *n* = 168 and 105; 1 or 2 mg BID; 12 wk	MWT: +13.8 to +20.1 min. ESS: −8.0 to −9.8 points. Cataplexy: WCR IRR 0.25–0.38.	Insomnia 55–60% vs. 1% placebo; urinary frequency 53–58% vs. 5%; CPK > 5 × ULN 11% vs. 5%	Approved; peer-reviewed + FDA label. Recognizable AEs may threaten masking of subjective outcomes; controlled exposure 12 wk [32,33]
Alixorexton (ALKS 2680)	Randomized phase 2 Vibrance-1 NT1; *n* = 92; 4/6/8 mg QD; 6 wk	MWT: +22.2/+24.1/+26.0 min. ESS: −6.4/−8.7/−8.3 points. Cataplexy: WCR rate ratios 0.49/0.31/0.64; only 6 mg statistically significant.	Any TEAE 87–91% active vs. 48% placebo; pollakiuria 50–65% vs. 4%; insomnia 17–33% vs. 0%	Peer-reviewed NT1 evidence; 6 wk exposure, so numerical comparison with 8- or 12 wk trials is not valid [46]
Cleminorexton (ORX750)	Registrational phase 2/3; NT1/NT2; oral	MWT/ESS/cataplexy: no mature peer-reviewed quantitative patient dataset.	Long-term safety not established	Development-stage evidence from primary registry; no comparative efficacy inference [49]
Ledasorexton (E2086)	Phase 2 narcolepsy program; oral	MWT/ESS/cataplexy: no mature peer-reviewed quantitative patient dataset.	Long-term safety not established	Investigational; registry/sponsor development-stage evidence [50]
TAK-360	Phase 2 NT2/IH development; oral	MWT/ESS/cataplexy: no mature peer-reviewed quantitative patient dataset.	Long-term safety not established	Primary registry evidence; no comparative efficacy inference [51,52]
ORX142	Phase 1 first-in-human; healthy adults; oral	MWT/ESS/cataplexy: no mature patient efficacy dataset.	Safety/PK under evaluation	Early-stage primary registry evidence; includes sleep-deprivation proof-of-concept component [53]
Lu AH69593	Phase 1/1b clinical program; healthy adults/NT1; oral	MWT/ESS/cataplexy: no mature peer-reviewed quantitative patient dataset.	Safety/PK under evaluation	Sponsor describes phase 1b development; early-stage evidence [54]
KSP-0930	Randomized phase 1; healthy adults; oral	MWT/ESS/cataplexy: no mature patient efficacy dataset.	Safety/PK/PD under evaluation	Early-stage primary registry evidence [55]

Cross-agent values are descriptive and should not be interpreted as head-to-head or indirect comparative efficacy estimates. Trials differ in population, dose, exposure duration (acute, 6, 8, or 12 weeks), endpoint timing, analytic methods, missing-data handling, and evidence maturity. Placebo comparisons and denominators are shown where mature data are available; NR indicates not reported or not yet available as a mature patient-level efficacy endpoint. Abbreviations: AE, adverse event; BID, twice daily; CPK, creatine phosphokinase; DILI, drug-induced liver injury; ESS, Epworth Sleepiness Scale; IH, idiopathic hypersomnia; IRR, incidence-rate ratio; IV, intravenous; MWT, Maintenance of Wakefulness Test; NR, not reported/not mature; NT1/NT2, narcolepsy type 1/2; QD, once daily; WCR, weekly cataplexy rate.

## Data Availability

No new data were created or analyzed in this study. Data sharing is not applicable to this article.
